# A multi-center international study to evaluate the safety, functional and oncological outcomes of irreversible electroporation for the ablation of prostate cancer

**DOI:** 10.1038/s41391-023-00783-y

**Published:** 2024-01-09

**Authors:** Kai Zhang, Phillip Stricker, Martin Löhr, Michael Stehling, Michel Suberville, Olivier Cussenot, Luca Lunelli, Chi-Fai Ng, Jeremy Teoh, Pilar Laguna, Jean de la Rosette

**Affiliations:** 1 Department of Urology, Beijing United Family Hospital and Clinics, Beijing, China; 2 Department of Urology, St Vincents Private Hospital Sydney, St Vincents Prostate Cancer Research Centre and The Garvan Institute, Sydney, Australia; 3 Department of Urology, Klinik für Prostata-Therapie, Heidelberg, Germany; 4 VITUS Privatklinik and Institut für Bildgebende Diagnostik, Strahlenbergerstrasse, Offenbach, Germany; 5 Doctor Michel SUBERVILLE Chief of Pôle, Pôle SAINT GERMAIN - CENTRE HOSPITALIER, Brive la Gaillarde, France; 6 Department of Urology, Tenon Hospital, Paris, France; 7 Department of Urology, Hospital Louis Pasteur, Chartres, France; 8 S.H. Ho Urology Centre, Department of Surgery, The Chinese University of Hong Kong, Hong Kong, China; 9 Department of Urology, Medipol Mega hospital, Istanbul Medipol University, Istanbul, Türkiye

**Keywords:** Prostate cancer, Outcomes research

## Abstract

**Background:**

Irreversible electroporation (IRE) is a novel technique to treat localized prostate cancer with the aim of achieving oncological control while reducing related side effects. We present the outcomes of localized prostate cancer treated with IRE from a multi-center prospective registry.

**Methods:**

Men with histologically confirmed prostate cancer were recruited to receive IRE. All the patients were proposed for prostate biopsy at 1-year post-IRE ablation. The functional outcomes were measured by the International Prostate Symptom Score (IPSS) and International Index of Erectile Function (IIEF-5) questionnaires. The safety of IRE was graded by the treatment-related adverse events (AEs) according to the Common Terminology Criteria for Adverse Events (CTCAE).

**Results:**

411 patients were recruited in this study from July 2015 to April 2020. The median follow-up time was 24 months (IQR 15–36). 116 patients underwent repeat prostate biopsy during 12–18 months after IRE. Clinically significant prostate cancer (Gleason ≥ 3 + 4) was detected in 24.1% (28/116) of the patients; any grade prostate cancers were found in 59.5% (69/116) of the patients. The IPSS score increased significantly from 7.1 to 8.2 (*p* = 0.015) at 3 months but decreased to 6.1 at 6 months (*p* = 0.017). Afterwards, the IPSS level remained stable during follow-up. The IIEF-5 score decreased at 3 months from 16.0 to 12.1 (*p* < 0.001) and then maintained equable afterwards. The rate of AEs was 1.8% at 3 months and then dropped to less than 1% at 6 months and remained stable until 48 months after IRE. Major AEs (Grade 3 or above) were rare.

**Conclusion:**

For men with localized prostate cancer, IRE could achieve good urinary and sexual function outcomes and a reasonable oncological result. The real-world data are consistent with earlier studies, including recently published randomized controlled studies. The long-term oncological results need further investigation and follow-up.

## Introduction

Radical treatment for localized prostate cancer often confers a major impact on quality of life [1, 2]. This has led to the development of focal therapy for prostate cancer, aiming to achieve equivalent oncological control whilst improving urinary and erectile function preservation.

Irreversible electroporation (IRE) is a novel technique using pulsed high-voltage low-energy direct electric current for tumor ablation. As a non-thermal energy platform, IRE has the advantage of sparing surrounding functional structures, including blood vessels and connective tissue [3, 4]. Histopathological outcomes after IRE show a sharp demarcation between ablated and non-ablated tissue, whereas thermal ablation techniques show a transitional zone of partially damaged tissue due to insufficient temperatures for definitive ablation [5].

This study presents the results from a multi-center prospective registry, reporting the IRE ablation therapy for localized prostate cancer patients regarding the side effects, quality of life and oncological control in a short- to mid-term follow-up.

## Patients and methods

### Study design

This was an international prospective observational multi-center study. The study was conducted according to good clinical practices and the Declaration of Helsinki and was approved by the IRB of the participating centers. The protocol is registered with the clinicaltrials.gov database (NCT02255890).

### Patient selection

The study population comprised patients with histologically confirmed prostate cancer who were treated with IRE. In order to capture real-world data, there were no specific exclusion criteria in our protocol. Informed consent was taken before receiving IRE.

### Treatment protocol

The AngioDynamics Inc. NanoKnife™ System was used to deliver IRE in this study. Transperineal or transrectal mapping prostate biopsy was performed using ultrasound guidance to diagnose and localize the prostate cancer.

Patients received a mpMRI scan pre-operatively, and the MRI imaging data was entered into the NanoKnife planning software. The volume and shape of the prostate ablation zone were then determined.

All patients received antibiotic prophylaxis prior to the procedure. A maximum of six IRE electrodes were placed into the pre-specified ablation zone using biplane transrectal ultrasound image guidance to visualize both sagittal and axial views. 90 consecutive high-voltage pulses (1500 V/cm) with a direct current between 20 and 50 A were delivered. During the IRE procedure, patients received muscle relaxants to prevent severe muscle contraction. The whole procedure was performed under general anesthesia, and patients were expected to stay overnight for observation and discharged the next day.

### Study outcomes

The primary outcome was the recurrence rate of prostate cancer at 1 year. The patients were proposed for prostate biopsy at 1-year post-IRE ablation. The recurrence was defined as persistent prostate cancer detected in the repeat biopsy. Secondary outcomes included functional outcomes as measured by the International Prostate Symptom Score (IPSS) and International Index of Erectile Function (IIEF-5) questionnaires, and the safety profile of IRE, as graded by the treatment-related adverse events (AEs) according to the Common Terminology Criteria for Adverse Events (CTCAE).

### Data collection and follow-up

All related data, including procedure records, adverse events and questionnaires, were collected, and patients were followed up every 3 months in the first 2 years, every 6 months in the third year and subsequently every 12 months, for up to 60 months post-IRE.

Data from participating centers will be collected through electronic Case Report Forms (eCRFs), with the use of an online Data Management System (DMS), which is located and maintained at the Clinical Research Office of The Endourological Society (CROES) Office. All statistical analyses will be performed by members of CROES Office. An in-person and/or remote audit was performed for quality assurance.

### Statistical analysis

Descriptive statistics were used to present the different parameters during follow-up. Changes from baseline for IPSS, IIEF-5 were compared at each follow-up visit using paired t-test with the baseline score as a continuous variate. A two-sided *p*-value of <0.05 was considered statistically significant. Statistical analyses were performed using SPSS for Windows (Version 27, IBM Corp Armonk, NY, USA).

## Results

### Baseline characteristics

From July 2015 to April 2020, 411 patients were recruited in this study. The median follow-up time was 24 months (IQR 15–36). The demographics and baseline characteristics are presented in Table 1.

**Table 1:** Baseline characteristics, perioperative outcomes of the patients.

Characteristics	IRE cohort (411)
Age, mean (SD), years	67.3 ± 7.4
Prostate volume, mean (SD), ml	44.3 ± 21.2
PSA, median (IQR), ng/ml	6.3 (4.4–9.7)
Number of biopsy cores, median (IQR)	13 (12–20)
Positive cores, median (IQR)	3 (2–5)
ISUP, No. (%)	
1	126 (30.7)
2	193 (47.1)
3	58 (14.1)
4	12 (2,9)
5	17 (4.1)
T stage, No. (%)	
cT1c	182 (46.8)
cT2a	101 (26.0)
cT2b	37 (9.5)
cT2c	65 (16.7)
cT3a	3 (0.8)
cT3b	1 (0.3)
IPSS, mean (SD),	7.7 ± 6.5
IIEF-5, mean (SD),	17.0 ± 7.8
Operative time, median (IQR), min	45 (30–70)
Number of IRE electrodes, median (IQR)	4 (4–5)
Duration of indwelling catheter, median (IQR), hour	48 (18–120)

T stage was based on the MRI findings prior to IRE.

*ISUP* International Society of Urological Pathology, *IPSS* International Prostate Symptom Score, *IIEF* The International Index of Erectile Function.

### Early oncological control

A total of 116 patients underwent repeat prostate biopsy during 12–18 months after IRE. Among the patients who underwent repeat prostate biopsy, clinically significant prostate cancer (Gleason ≥ 3 + 4) was detected in 24.1% (28/116) of them; any grade prostate cancers were found in 59.5% (69/116) of the patients. Among 38 patients with clinically insignificant prostate cancer (Gleason ≤ 3 + 3) in the initial biopsy, 18 cases of insignificant tumor and 8 cases of significant tumor were detected in the repeat biopsy. Among 76 patients with clinically significant cancer in the initial biopsy, 23 cases of insignificant tumor and 20 cases of significant tumor were detected in the repeat biopsy (*p* = 0.156). The results are summarized in Table 2 and Supplementary Tables 1 and 2.

**Table 2:** The repeat biopsy results at the 12–18 months post-operation.

Prostate biopsy	IRE cohort (116)
Clinically significant prostate cancer	28 (24.1%)
Any grade prostate cancer	69 (59.5%)
ISUP, No. (%)	
1	41 (35.3%)
2	15 (12.9%)
3	4 (3.4%)
4	5 (4.3%)
5	4 (3.4%)

*ISUP* International Society of Urological Pathology.

**Fig. 1:**
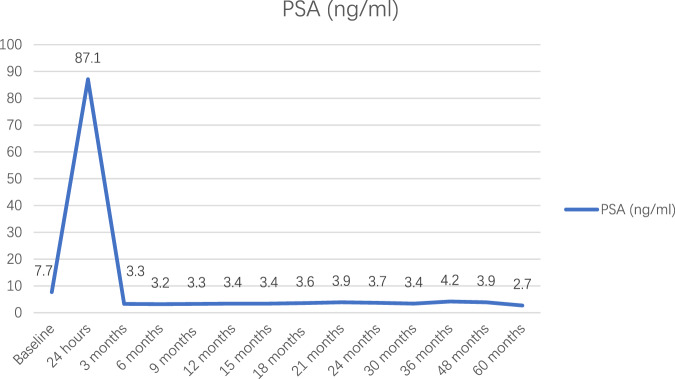
The change of PSA values. The PSA values at baseline, 24 hours post-operation and each follow-up point.

The PSA value rose significantly in the first 24 h post-operation and dropped sharply by 96.2% in the subsequent 3 months. Afterwards, the values were lower than the baseline level and remained equable (Fig. 1). The PSA level of patients with positive biopsy was significantly higher than those with negative biopsy (5.2 ng/ml vs 2.7 ng/ml, *p* < 0.01).

Among the 69 patients who had positive results in the repeat biopsy, 27 patients underwent salvage treatment, including 16 cases of clinically significant cancer and 7 cases of insignificant cancer. 5 patients underwent androgen deprivation therapy (ADT) afterwards, 8 patients underwent IRE, 9 patients underwent prostatectomy, 3 patients underwent radiation therapy, 1 patient underwent transurethral resection of the prostate, 1 patient underwent ADT combined with IRE.

### Functional outcomes

At 3-month follow-up, the IPSS score increased significantly from 7.1 to 8.2 (*p* = 0.015) when compared to the baseline level. It went down to 6.1 and achieved an even lower value than the baseline level at 6-months (*p* = 0.017). Afterwards, the IPSS level remained stable during follow-up.

The IIEF-5 score dropped at 3-months from 16.0 to 12.1 (*p* < 0.001). Although the IIEF level was statistically lower at each follow-up point than the baseline level, it maintained equable after 3 months. The results of the functional outcomes are summarized in Table 3.

**Table 3:** The change of quality of life from baseline through follow-up.

	3 months	6 months	9 months	12 months	18 months	24 months	30 months
IPSS							
*n*, paired values	215	160	123	117	72	63	42
Baseline	7.1 ± 5.8	7.1 ± 5.9	7.7 ± 5.9	7.4 ± 6.0	7.7 ± 6.4	8.5 ± 6.7	8.6 ± 6.3
Follow-up	8.2 ± 5.9	6.1 ± 5.5	7.2 ± 6.5	6.4 ± 5.0	7.8 ± 6.4	8.1 ± 5.6	8.7 ± 8.2
Change	1.1	−1.0	−0.5	−1.1	0.2	−0.3	0.1
95% CI	0.2 to 1.9	−0.2 to −1.8	−1.5 to 0.6	−0.04 to −2.1	−1.2 to 1.5	−2.0 to 1.2	−2.2 to 2.5
*p* value	0.015	0.017	0.41	0.04	0.82	0.65	0.90
IIEF-5							
*n*, paired values	139	124	95	89	55	36	28
Baseline	16.0 ± 8.6	15.7 ± 8.6	14.9 ± 8.6	15.3 ± 8.6	14.4 ± 8.6	16.0 ± 9.0	15.0 ± 9.1
Follow-up	12.1 ± 8.7	11.5 ± 8.4	11.5 ± 8.3	11.2 ± 8.5	10.8 ± 8.5	12.3 ± 9.0	10.9 ± 9.1
Change	−3.8	−4.2	−3.4	−4.0	−3.7	−3.8	−4.1
95% CI	−4.9 to −2.7	−5.4 to −3.1	−4.7 to −2.0	−5.5 to −2.6	−5.6 to −1.7	−6.9 to −0.6	−7.6 to −0.5
*p* value	<0.001	<0.001	<0.001	<0.001	<0.001	0.02	0.03

*IPSS* International Prostate Symptom Score, *QOL* Quality of Life, *IIEF* The International Index of Erectile Function, *CI* Confidence Interval.

When potency was defined as erections firm enough for intercourse more than half of the time (IIEF-5 Q2 ≥ 3), among patients who were potent before treatment, 71.0%, 68.8%, 74.1%, 84.3%, 72.4%, and 81.8% of men still had potency at 3, 6, 9, 12, 18 and 24 months respectively.

### Adverse events

The rate of AEs was the highest at 3-month after IRE, with 1.8% in the whole group. The rates of AEs dropped to less than 1% at 6-month and remained similar until 48 months after IRE. Major AEs (Grade 3 or above) were rare; there were two cases at 3-month and one case at 15-month. The relationship between AEs and IRE was classified as definitely, probably, possibly, unlikely, and not related. Among all the 13 AEs, 46.2% of cases were identified as “not related” and only one major AE was identified as “definitely”. The results on AEs are summarized in Table 4.

**Table 4:** The adverse events of patients during follow-up.

Follow-up		IRE group	Relationship with the procedure
3 months	AE	7/383 (1.8%)	
	AE Grade		
	1	2	Not related/Probably
	2	3	2 Probably/ Possibly
	3	1	Definitely
	4	1	Not related
6 months	AE	2/299 (0.67%)	
	AE Grade		
	1	1	Probably
	2	1	Not related
9 months	AE	1/240 (0.42%)	
	AE Grade		
	1	1	Not related
12 months	AE	0/264	
15 months	AE	1/175 (0.57%)	
	AE Grade		
	3	1	Not related
18 months	AE	0/155	
21 months	AE	0/118	
24 months	AE	1/158 (0.63%)	
	AE Grade		
	2	1	Definitely
30 months	AE	0/122	
36 months	AE	1/99 (1.0%)	
	AE Grade		
	2	1	Not related
48 months	AE	0/48	

*IAE* adverse events.

## Discussion

In our study, IRE achieved good functional results and promising oncological results. The IPSS score worsened at 3 months but resumed to baseline at 6 months. The IIEF-5 score worsened at 3 months and remained stable afterwards. The AEs rate of IRE was very low, only 1.8% at 3 months and less than 1% subsequently. In the repeat biopsy 12–18 months post-IRE, clinically significant prostate cancer was detected in 24.1% of the patients.

Some studies have already showed excellent functional results post-IRE [6, 7]. In a recently published Chinese study, 117 patients underwent IRE, the median IPSS was 9.0 at baseline and 4.5 at 6 months; the median IIEF-5 was 2.0 at baseline and 2.0 at 6 months [8]. In a European study including 123 patients, the urinary function declined at 6 weeks after IRE, but recovered to baseline after 3 months. There was a mild but significant decrease in sexual function. Among patients who were potent before treatment, 76% had no change in potency at 12 months, 17% had erections firm enough for some sexual activity, and 7% did not have erections firm enough for any sexual activity [9]. Our study showed similar functional results.

Focal therapy was developed as an alternative to minimize adverse effects while maintaining a good oncological outcome for the treatment of prostate cancer. In the past decades, different energy sources in focal therapy have been studied [10]. IRE might have potential advantages over other focal therapy modalities as it is a non-thermal energy source and has an advantage of sparing surrounding vital structures such as blood vessels and nerve bundles [11]. In a prospective phase I/II study, 16 patients underwent IRE prior to radical prostatectomy; although the study showed that IRE effects were observed extending beyond the prostatic capsule and in the neurovascular bundle in most cases, chronic inflammation varied from mild to moderate with only one cases showing focal severe inflammation [5]. In an animal study using a rabbit model, the femoral nerve function was found to be damaged at 4-week post-IRE, but gradually returned to normal at 8-week [12]. Their findings were consistent with the clinical study results that IRE only caused a minimal effect on functional outcomes in patients even receiving extended focal ablation for prostate cancer treatment [13].

IRE showed excellent safety profile peri-operatively and upon follow-up. Within 12 months after the procedure, the overall AE rate was only 2.6% based on the CTCAE system. In the Chinese study, the overall complication rate was 37.6% during the 6-month follow-up. However, only one Clavien-Dindo grade 3 complication occurred [8]. The most common complication was elevated white blood cell level in urine (23.9%), followed by epididymitis (4.6%), prolonged gross hematuria (3.7%), urinary retention (2.8%), urinary tract infection (1.8%) and bladder stones (0.9%) [8]. The European study demonstrated that the Clavien-Dindo grade 1 complication rate was 22%, including dysuria, urgency, hematuria, and perineal pain. 9% of patients experienced grade 2 complications, including urinary tract infection, severe urgency/frequency, or incontinence. There was no grade 3 or above complications being reported. No perioperative complications were recorded [9].

In our study, IRE revealed promising oncological results. In the repeat biopsy, the positive rate of clinically significant prostate cancer was 24.1%. The second focal therapy, or radical therapy, was still available for these patients. It is important to note that our study was performed in the initial stage of IRE technique worldwide. It was not standardized yet and the fusion biopsy technique and treatment recording was rarely available at that time. The oncologic results might be underestimated in our findings. In the European study, the significant prostate cancer rate was 22.5% at 12-month after IRE. The in-field recurrence was 9.8% and the out-of-field recurrence was 12.7% [9]. In a recently published randomized controlled study, clinically significant cancer found in the treated area was 9.9% in the repeat biopsy [14]. For the other types of focal therapy, significant prostate cancer in-field recurrence rate was described with a median of 14.7% for high-intensity focused ultrasound, 8.5% for IRE, 10% for photodynamic therapy, 15% for cryoablation, 17% for focal laser ablation, 20% for radiofrequency ablation, and 60% for prostatic artery embolization [15]. In one study including 229 patients with a median follow-up of 60 months, the failure-free survival was 91% at 3 years, 84% at 5 years and 69% at 8 years. The metastasis-free survival was 99.6% and the prostate cancer specific and overall survival were 100% [6].

Another important finding in the current study was that the recurrence rate was not associated with the ISUP grade at baseline, suggesting that the extension of the IRE indication might be safe. From 1996–2015, 76% of patients treated with focal therapy had Gleason 6 tumor. However, from 2015–2020, 51% of patients who underwent focal therapy had Gleason 7 tumor, with a stable small proportion of men with Gleason 8 tumor [10, 15]. Considering the high chance of conversion to radical treatment for patients with active surveillance and the minimal side effect of IRE [16], patients with low- to intermediate-risk prostate cancer who would be candidates for active surveillance may be more suitable for IRE treatment. A randomized trial displayed that conversion to radical treatment was less likely in the focal therapy group than in the active surveillance group [17].

There are some limitations that should be discussed. First, we had a considerable loss to follow-up in our study. At baseline, there were 411 patients, and only 28.2% of patients attended repeat biopsies during 12–18 months after IRE. Some biopsies were omitted because no suspicious lesions were found in the MRI scan post-IRE. The real recurrence rate may be overestimated in our study. The data on functional and oncological results were largely incomplete, with some key message missed, and the follow-up time was short. However, this is reflective of the management of prostate cancer in a real-world setting. The results from this real-world data are consistent with the randomized controlled studies using very strict protocols [13, 14]. Second, the template biopsy modality was applied in the repeat biopsy. Recording of the treatment zone is not possible at the time of the study, and we are not able to determine whether or not the positive cores were in the treated areas. It is difficult to compare our results to the other studies. Moreover, the use of MRI was limited in our study. 25% of the patients underwent prostate mapping biopsy without a MRI scan in the repeat biopsy. For a long time, imaging has been one of the major limitations of focal therapy. Although MRI significantly improved the clinically significant prostate cancer detection [18], the standardization for acquisition and MRI reporting in the post-focal therapy setting is still lacking [19]. The recurrence in the untreated areas increased the conversion from focal therapy to radical treatment and harmed the efficacy of focal therapy. In the future, IRE/focal therapy could benefit from improvements in prostate imaging by the potential use of artificial intelligence, radiomics, and other modalities such as positron emission tomography [20].

## Conclusions

IRE could achieve good urinary and sexual function outcomes in men with localized prostate cancer. The rate of AEs was 1.8% at 3 months after IRE and major AEs were rare. The IPSS initially worsened but returned to baseline level at 6 months. The IIEF-5 worsened at 3 months and then maintained stable afterwards. IRE was able to achieve a reasonable oncological outcome. The clinically significant prostate cancer rate in the repeat biopsy during 12–18 months was in 24.1%. The long-term oncological results need further investigation and follow-up. The real-world data are consistent with earlier studies including recently published randomized controlled studies.

### Supplementary information

Supplementary materials table 1

Supplementary materials table 2

## Data Availability

The datasets generated and/or analyzed during the current study are available from the corresponding author on reasonable request.
