# Supplementary material for: A multi-center international study to evaluate the safety, functional and oncological outcomes of irreversible electroporation for the ablation of prostate cancer
Source: Prostate Cancer Prostatic Dis. 2024 Jan 9;27(3):525–30. doi: 10.1038/s41391-023-00783-y (PMC11319192; doi:10.1038/s41391-023-00783-y)
Supplement: Supplementary file 1 — Supplementary materials table 1 [file 41391_2023_783_MOESM1_ESM.docx]

Supplementary Table 1: The change of biopsy results after treatment

| Biopsy**  Biopsy* | Negative | Clinically insignificant | Clinically significant | Total |
| --- | --- | --- | --- | --- |
| Clinically insignificant | 12 | 18 | 8 | 38 |
| Clinically significant | 33 | 23 | 20 | 76 |
| Total | 45 | 41 | 28 | 114 |

Biopsy*: Biopsy result in the baseline biopsy

Biopsy**: Biopsy results in the repeat biopsy
