# Supplementary material for: A multi-center international study to evaluate the safety, functional and oncological outcomes of irreversible electroporation for the ablation of prostate cancer
Source: Prostate Cancer Prostatic Dis. 2024 Jan 9;27(3):525–30. doi: 10.1038/s41391-023-00783-y (PMC11319192; doi:10.1038/s41391-023-00783-y)
Supplement: Supplementary file 2 — Supplementary materials table 2 [file 41391_2023_783_MOESM2_ESM.docx]

Supplementary Table 2 Repeat biopsy results in different centers

| Center (Patient number) | MRI post-IRE | Repeat biopsy | Any grade prostate cancer | Clinically significant prostate cancer | Clinically insignificant prostate cancer |
| --- | --- | --- | --- | --- | --- |
| Klinik for Prostata Therapie (83) | 47 | 6 | 4 | 2 | 2 |
| St Vincents Private Hospital Sydney (116) | 116 | 52 | 28 | 13 | 15 |
| Tenon Hospital (97) | 75 | 22 | 9 | 5 | 4 |
| Vitus Privatklinik and Institute for Bildgebende Diagnostik (53) | 37 | 3 | 2 | 2 | 0 |
| Clinique Mutualiste Saint Germain (61) | 54 | 33 | 26 | 6 | 20 |
